# Depressive symptoms, but not anxiety, predict subsequent diagnosis of Coronavirus disease 19: a national cohort study

**DOI:** 10.1017/S2045796021000676

**Published:** 2022-03-25

**Authors:** G. Meinlschmidt, S. Guemghar, N. Roemmel, E. Battegay, S. Hunziker, R. Schaefert

**Affiliations:** 1 Department of Psychosomatic Medicine, University Hospital Basel, Basel, Switzerland; 2 Faculty of Medicine, University of Basel, Basel, Switzerland; 3 Division of Clinical Psychology and Cognitive Behavioural Therapy, International Psychoanalytic University, Berlin, Germany; 4 Division of Clinical Psychology and Epidemiology, Department of Psychology, University of Basel, Basel, Switzerland; 5 International Center for Multimorbidity and Complexity in Medicine (ICMC), University of Zurich, Zurich, Switzerland; 6 Merian Iselin Clinic, Basel, Switzerland; 7 Medical Communication/Psychosomatic Medicine, University Hospital Basel, Basel, Switzerland

**Keywords:** Anxiety, comorbidity, corona, COVID-19, depression, mental disorders, multimorbidity, SARS-CoV-2

## Abstract

**Aims:**

Several diseases are linked to increased risk of Coronavirus disease 19 (COVID-19). Our aim was to investigate whether depressive and anxiety symptoms predict subsequent risk of COVID-19, as has been shown for other respiratory infections.

**Methods:**

We based our analysis on UK Biobank participants providing prospective data to estimate temporal association between depressive and anxiety symptoms and COVID-19. We estimated whether the magnitude of these symptoms predicts subsequent diagnosis of COVID-19 in this sample. Further, we evaluated whether depressive and anxiety symptoms predicted (i) being tested for severe acute respiratory syndrome coronavirus 2 (SARS-CoV-2) and (ii) COVID-19 in those tested.

**Results:**

Based on data from *N* = 135 102 participants, depressive symptoms (odds ratio (OR) = 1.052; 95% confidence interval (CI) 1.017–1.086; absolute case risk: (moderately) severe depression: 493 per 100 000 *v*. minimal depression: 231 per 100 000) but not anxiety (OR = 1.009; 95% CI 0.97–1.047) predicted COVID-19. While depressive symptoms but not anxiety predicted (i) being tested for SARS-CoV-2 (OR = 1.039; 95% CI 1.029–1.05 and OR = 0.99; 95% CI 0.978–1.002), (ii) neither predicted COVID-19 in those tested (OR = 1.015; 95% CI 0.981–1.05 and OR = 1.021; 95% CI 0.981–1.061). Results remained stable after adjusting for sociodemographic characteristics, multimorbidity and behavioural factors.

**Conclusions:**

Depressive symptoms were associated with a higher risk of COVID-19 diagnosis, irrespective of multimorbidities. Potential underlying mechanisms to be elucidated include risk behaviour, symptom perception, healthcare use, testing likelihood, viral exposure, immune function and disease progress. Our findings highlight the relevance of mental processes in the context of COVID-19.

## Introduction

Severe acute respiratory syndrome coronavirus 2 (SARS-CoV-2) causes Coronavirus disease 19 (COVID-19) and has spread across the globe, with over 23 million confirmed cases worldwide as of 24 August 2020 (the date of the latest data available for this study; World Health Organization, 2020). The COVID-19 pandemic has impacted mortality and entire societies substantially (Petterson *et al*., 2020; World Health Organization, 2020). Several conditions, diseases and sociodemographic factors have been associated with COVID-19 and related deaths (Richardson *et al*., 2020; Rozenfeld *et al*., 2020; Wang *et al*., 2020*a*; Williamson *et al*., 2020; Zhou *et al*., 2020). Recent population-based, prospective studies found evidence for an association between the history of depression or anxiety and the subsequent risk of COVID-19 (Lee *et al*., 2020; Li *et al*., 2020*b*; Wang *et al*., 2021; Fond *et al*., 2021). However, the relevance of depression or anxiety to the likelihood of being tested for SARS-CoV-2, additionally to being diagnosed with COVID-19 remains unclear.

Notably, subjects with COVID-19 show increased rates of psychosocial stress, including depressive and anxiety symptoms, as well as disturbed sleep (Krishnamoorthy *et al*., 2020; Li *et al*., 2020*a*; Mazza *et al*., 2020; Rogers *et al*., 2020). However, given the cross-sectional nature of previous studies, studies need to determine the directions of these associations. Indeed, the psychosocial burden of COVID-19 and its symptoms, potentially severe or fatal disease trajectories, treatment and required isolation may trigger these symptoms. On the other hand, depression and anxiety may precede respiratory diseases (Goodwin *et al*., 2014). Indeed, multiple studies have shown a link between mental factors and infectious diseases, including respiratory tract infections (Goodwin *et al*., 2003; Adam *et al*., 2013). However, it is unknown whether symptoms of mental disorders precede and predict the subsequent risk of COVID-19.

The objective of our study was to evaluate the association of depressive symptoms and anxiety with the subsequent risk of being diagnosed with COVID-19. We hypothesised that the magnitudes of both are linked to an increased risk of COVID-19. Further, given the potential relevance of testing rates and their relation to mental disorders (van der Meer *et al*., 2020), we estimated associations between depressive and anxiety symptoms with the likelihood of being tested for SARS-CoV-2. Scrutinising the role of mental factors for disease trajectories of COVID-19 in the UK Biobank, a large-scale study that includes prospective data may contribute to a better understanding and potentially better management of COVID-19.

## Methods

### Study design and population

The present study is based on the UK Biobank, a large population-based national cohort of UK residents (Smith *et al*., 2013). Voluntary participants were recruited between March 2006 and December 2010. Additionally, a proportion of participants were invited to repeat assessments and to answer questionnaires between 2012 and 2019. During these subsequent visits, some information that was missing at the initial assessment was collected. Further, information from the hospital inpatient data was linked to the UK Biobank dataset, cancer register, death register and primary care data. Information provided by participants at recruitment and at subsequent assessments included sociodemographic characteristics, self-reported health conditions and answers to a mental health web-based questionnaire between 2016 and 2017. We used the latter to calculate the nine-item Patient Health Questionnaire (PHQ-9) (Spitzer, 1999; Spitzer *et al*., 2000; Kroenke *et al*., 2001; Kroenke and Spitzer, 2002) and seven-item Generalised Anxiety Disorder (GAD-7) (Spitzer *et al*., 2006; Löwe *et al*., 2008; Dear *et al*., 2011) scores where possible. Information on SARS-CoV-2 tests of UK Biobank participants was provided by Public Health England (PHE) for the period from 16 March 2020 to 24 August 2020. We included participants who were recruited in England, were alive on 31 December 2019 before the onset of the COVID-19 pandemic, and provided sufficient information on PHQ-9 and GAD-7 available so that we were able to calculate respective scores. We provide more details on the UK Biobank and the study procedures in online Supplementary material 1.

### Statistical analysis

To estimate the association of depressive and anxiety symptoms with COVID-19, we conducted logistic regression analyses. For the crude models, we entered depressive and anxiety symptom scores as continuous predictor variables with COVID-19 as the main outcome. For adjusted models, we conducted a two-step adjustment scheme. As a first step, we concomitantly adjusted the analyses for *a priori* defined sociodemographic variables, acting as potential confounders: age, sex, ethnicity and deprivation index, categorised as outlined in Table 1. As a second step, we adjusted analyses for *a priori* selected physical diseases and behavioural risk factors (see Table 1) that have been reported elsewhere as being linked to an increased risk of COVID-19 (Wang *et al*., 2020*a*; Zhou *et al*., 2020). These may potentially act as confounders or mediators, given the lack of information on the timing of these physical diseases and behavioural risk factors as compared to the depressive and anxiety symptoms. For the second adjustment step, we entered first step covariates, as well as one physical disease or behavioural risk factor at a time. Next, to estimate the association between depressive and anxiety symptoms with *being tested for SARS-CoV-2*, we conducted additional logistic regression analyses. Here again, we applied the two-step adjustment scheme outlined above. Further, to estimate the association of depressive and anxiety symptoms with COVID-19 *in those being tested for SARS-CoV-2*, we conducted logistic regression analyses as outlined above, this time however restricting the analyses to participants who had been tested for SARS-CoV-2. To prevent overfitting, we adjusted for only one sociodemographic variable at a time in step 1 and omitted adjusting for step 1 covariates during step 2.

**Table 1.** Descriptive statistics of UK Biobank participants included in the study

	Total *N* = 135 102
	Frequency (%)^a^
*Sociodemographic information*	
Sex	
Female	76 457 (56.6%)
Male	58 645 (43.4%)
Age^b^ (years)	
Median (IQR) [range]	68 (61–73) [49–83]
Age intervals^b^ (years)	
49–54	11 313 (8.4%)
55–59	17 858 (13.2%)
60–64	21 973 (16.3%)
65–69	27 170 (20.1%)
70–74	33 589 (24.9%)
75–79	20 694 (15.3%)
80–83	2505 (1.9%)
Ethnicity^c^	
White	130 590 (96.7%)
Black	1059 (0.8%)
South Asian	1200 (0.9%)
Other	1836 (1.4%)
Townsend category^d^	
Least deprived <−2	75 890 (56.2%)
Average (−2, 2)	42 304 (31.3%)
Most deprived ≥2	16 740 (12.4%)
Died after 31 December 2019	502 (0.4%)
*Multimorbidities*	
Asthma	18 226 (13.5%)
Cancer	20 815 (15.4%)
Cerebrovascular disease	2571 (1.9%)
COPD	2907 (2.2%)
Coronary artery disease	6927 (5.1%)
Diabetes mellitus^e^	6664 (4.9%)
Hypertension	24 114 (17.8%)
Body mass index^f^ (kg/m^2^)	
Median (IQR) [range]	26 (24–29) [12–70]
Obesity (BMI ≥ 30)	26 465 (19.6%)
Morbid obesity (BMI ≥ 35)	7070 (5.2%)
Never smoked^g^	77 187 (57.2%)
Never drank^h^	3630 (2.7%)

BMI, body mass index; COPD, chronic obstructive pulmonary disease; IQR, interquartile range.

a Percentages may not add up to 100 because of rounding.

b Age on 1 January 2020.

c White includes British, Irish and any other white background. Black includes Caribbean, African and any other black background. South Asian includes Indian, Pakistani, Bangladeshi and any other south Asian background. Other includes mixed, Chinese or other ethnicities. 417 participants were missing ethnicity data.

d Participants were assigned a Townsend deprivation score corresponding to the output area of their residential postcode). 168 participants were missing Townsend scores.

e Includes diet-controlled and non-insulin-dependent diabetes.

f 283 participants were missing BMI data.

g 266 participants were missing smoking status data.

h 107 participants were missing drinking status data.

We excluded participants who did not answer all the questions in the mental health web-based questionnaire necessary to calculate GAD-7 and PHQ-9 scores. We handled missing data by conducting completer analyses, excluding participants who lacked information on ethnicity, Townsend score, body mass index, or smoking and drinking status, when adjusting for these confounders, respectively (see online Supplementary Tables 4–8 in online Supplementary material 3).

To further test for a dose–response relationship, we conducted a logistic regression analysis with depressive symptom scores as ordered categorical predictor variables and COVID-19 as the outcome.

We provided estimates with 95% confidence intervals. We performed all calculations at sciCORE (sciCORE | Center for Scientific Computing, 2020) scientific computing centre at the University of Basel, using R version 4.0.0 (R Core Team, 2020). We provide more details on statistical analyses in online Supplementary material 2.

## Results

**Fig. 1.**
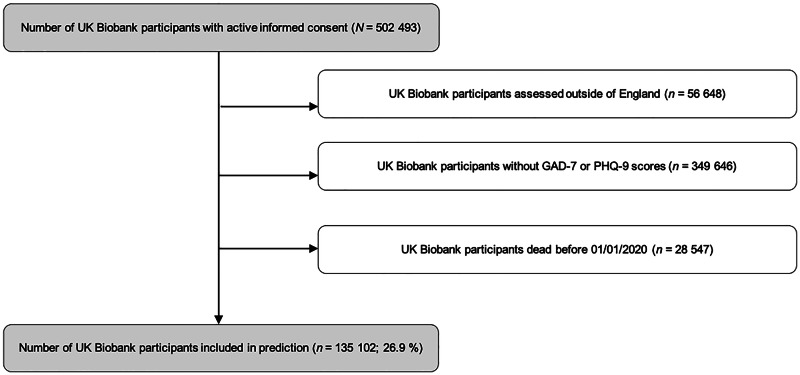
Flow chart of study participants. Predictions were calculated with UKB participants assessed in England, alive on 31 December 2019 and with complete GAD-7 and PHQ-9 scores.

Figure 1 shows the flow chart of study participants.

Table 1 shows descriptive information on sociodemographic characteristics, multimorbidities and behavioural factors in the 135 102 UK Biobank participants on which our analyses are based. In our sample, 3217 of these participants were tested for SARS-CoV-2. Of these, 337 tested positive.

Depressive symptoms but not anxiety symptoms predicted COVID-19 (OR = 1.052; 95% CI 1.017–1.086; *p* = 0.0024 and OR = 1.009; 95% CI 0.97–1.047; *p* = 0.65, respectively). Estimates remained stable when adjusting for potential sociodemographic confounders (OR = 1.037; 95% CI 1.002–1.072; *p* = 0.034 and OR = 1.005; 95% CI 0.965–1.044; *p* = 0.82, respectively), as well as when adjusting for individual physical diseases and behavioural factors (see online Supplementary Tables 4 and 5 in online Supplementary material 3).

Depressive symptoms but not anxiety symptoms predicted being tested for SARS-CoV-2 (OR = 1.039; 95% CI 1.029–1.05; *p* < 0.0001 and OR = 0.99; 95% CI 0.978–1.002; *p* = 0.08, respectively). Estimates remained stable when adjusting for potential sociodemographic confounders (OR = 1.042; CI 1.032–1.053; *p* < 0.0001 and OR = 0.993; CI 0.981–1.005; *p* = 0.24, respectively), as well as when additionally adjusting for individual physical diseases and behavioural factors (see online Supplementary Tables 6 and 7 in online Supplementary material 3).

Neither depressive symptoms nor anxiety symptoms predicted COVID-19 in those tested for SARS-CoV-2 in the crude models (OR = 1.015; 95% CI 0.981–1.05; *p* = 0.38 and OR = 1.021; 95% CI 0.981–1.061; *p* = 0.30, respectively) as well as in the adjusted models (see online Supplementary Table 8 in online Supplementary material 3).

Depressive symptoms have a dose–response effect on COVID-19 (OR = 1.77; 95% CI 1.16–2.55; *p* = 0.0041).

**Fig. 2.**
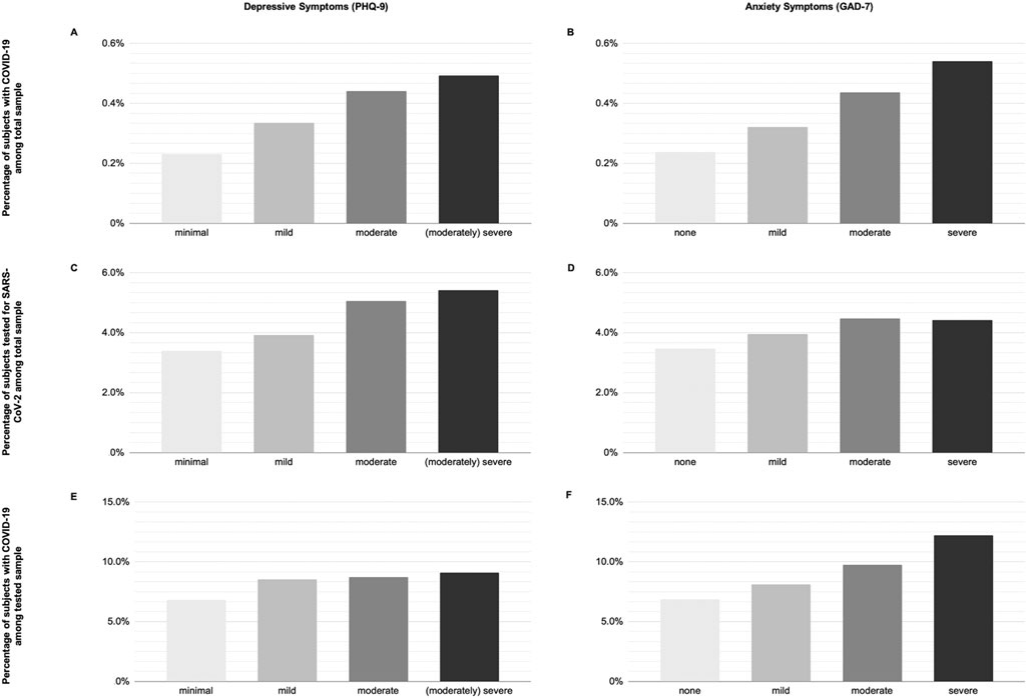
Percentage of study participants with COVID-19. (A and B) Percentage of subjects with COVID-19 in the total sample stratified by depressive symptoms (A) and general anxiety disorder (B). (C and D) Percentage of subjects tested for SARS-CoV-2 in the total sample stratified by depressive symptoms (C) and general anxiety disorder (D). (E and F) Percentage of subjects with COVID-19 in the tested sample stratified by depressive symptoms (E) and general anxiety disorder (F). *PHQ-9 score:* 0–4, minimal; 5–9, mild; 10–14, moderate; 15–27, (moderately) severe. *GAD-7 score:* 0–4, none; 5–9, mild; 10–14, moderate; 15–21, severe. COVID-19, Coronavirus disease 19; GAD-7 scale, Generalised Anxiety Disorder 7-item scale; PHQ-9, Patient Health Questionnaire Depression 9-item scale; SARS-CoV-2, severe acute respiratory syndrome coronavirus 2.

We depict the probabilities of COVID-19 in the total sample, being tested for SARS-CoV-2 in the total sample, and COVID-19 in those tested, stratified by depressive symptom and anxiety symptom severity categories in Fig. 2. Additionally, we estimated the unadjusted absolute risks (AR) and risk differences (RD) expressed as the number of cases per 100 000 subjects (see Table 2).

**Table 2.** Unadjusted absolute risks and risk differences with and without depression and anxiety of COVID-19 and being tested for SARS-CoV-2, in cases per 100 000 subjects^a^

	Depressive symptoms			Anxiety symptoms		
	Minimal	(Moderately) severe	(Moderately) severe – minimal	None	Severe	Severe – none
	AR	AR	RD	AR	AR	RD
COVID-19 in total sample	231	493	262	238	541	303
Being tested for SARS-CoV-2	3395	5419	2024	3464	4425	961
COVID-19 in tested sample	6805	9091	2286	6864	12 222	5358

AR, absolute risk; COVID-19, Coronavirus disease 19; RD, risk difference; SARS-CoV-2, severe acute respiratory syndrome coronavirus 2.

a Number of subjects rounded to the nearest digit.

## Discussion

Results of our population-based study, using prospective and self-report data provide evidence that the magnitude of depressive but not of anxiety symptoms years before the COVID-19 pandemic predicts being tested for SARS-CoV-2 and COVID-19 diagnosis. These results remained stable after adjusting for potential confounders including other comorbidities. In those tested for SARS-CoV-2, there were no further associations of depressive symptoms or anxiety with COVID-19. Notably, the magnitude of association between depressive symptoms and COVID-19 was comparable to associations between physical diseases and COVID-19, and remained stable after adjusting for multiple morbidities, known to predict risk of COVID-19. This is in line with previous population-based studies drawing on prospective data that found evidence for an association between the history of depression or anxiety and COVID-19 (Lee *et al*., 2020; Li *et al*., 2020*b*; Wang *et al*., 2021; Fond *et al*., 2021).

Our findings strongly support our hypothesis that the magnitude of depressive symptom severity precedes and predicts an increased risk of subsequently being diagnosed with COVID-19. To our surprise, symptoms of anxiety were not associated with the risk of subsequently being diagnosed with COVID-19 beyond depressive symptoms. Notably, by using population-based prospective data, allowing dose–response estimates, we extend, substantiate and specify evidence based on health records (Wang *et al*., 2020*a*, 2021). This indicates that being diagnosed with a mental disorder is linked to an increased risk of being diagnosed with COVID-19 (Taquet *et al*., 2021).

Our findings are in line with previous own (Adam *et al*., 2013) and others' studies (Goodwin *et al*., 2014) that indicate a link between symptoms of mental disorders and an increased risk of respiratory diseases, such as the common cold. Interestingly, seropositivity for other coronaviruses has been associated with a history of mood disorders (Okusaga *et al*., 2011). Our findings show that depressive symptoms predict the risk independently of previously described mental conditions, such as nicotine use, indicative of tobacco use disorder (Gülsen *et al*., 2020; Reddy *et al*., 2021).

Several potential mechanisms may explain our main finding of depressive symptoms predicting the risk of a confirmed COVID-19 diagnosis.

First, depressive symptoms are commonly associated with altered behavioural patterns that may be linked to the risk of infections, including hygiene measures, physical and social activities. More specifically, depressive symptoms were associated with reduced self-reported adherence with consistent wearing of face masks and self-reported sanitizing of hands (Pan *et al*., 2020). This in turn may increase the risk of being exposed to the SARS-CoV-2 virus and hence infections (Lin Huang *et al*., 2014; Wang *et al*., 2020*b*). However, reduced social activities may decrease the risk of COVID-19 infections as decreasing social interactions equally reduced the risk of infections (Wiersinga *et al*., 2020). Second, depressive symptoms are linked to impaired immune function increasing the risk of infection (Irwin and Miller, 2007; Dubois *et al*., 2017). Also, depressive symptoms are associated with increased levels of proinflammatory cytokines, C-reactive protein, leukocytes and neutrophil-to-lymphocyte ratio in COVID-19 patients and beyond, suggesting an increased prevalence of low-grade inflammation (Dowlati *et al*., 2010; Osimo *et al*., 2019; Yuan *et al*., 2020). This may contribute to exaggerated inflammatory responses to SARS-CoV-2 and subsequent tissue damage as well as more severe disease courses (Tay *et al*., 2020). Similarly, the immune system may senesce more quickly in subjects with mood disorder (Rizzo *et al*., 2018), which in turn may lead to more severe disease courses and detrimental outcomes of COVID-19 (Brietzke *et al*., 2020). Notably, a recent report indicates that antidepressants in the form of selective serotonin reuptake inhibitors may improve COVID-19 disease trajectories, highlighting the potential mechanistic relevance of depression for COVID-19 disease courses (Lenze *et al*., 2020).

Third, some subjects with depressive symptoms may be particularly concerned, and may therefore seek testing more readily if they suspect they have COVID-19; or they may seek access to healthcare as soon as they perceive symptoms, due to a heightened sensitivity to physical symptoms. Similarly, medical professionals may tend to test these subjects more frequently, leading to higher propensity of detecting SARS-CoV-2 infections. This is reflected by our finding that depressive symptoms are associated with higher likelihood of being tested. This is in line with previous reports that a clinical diagnosis of a mental disorder is associated with an increased likelihood of being tested for COVID-19 (van der Meer *et al*., 2020). Interestingly, our findings do not support the hypotheses that anxiety – beyond depression – is linked to an increased likelihood of being tested for SARS-CoV-2 or to an increased risk of a diagnosis of COVID-19. This is intriguing, because anxiety could either encourage seeking testing for reassurance or on the contrary discourage it for fear of a positive result.

Our study has important strengths. First, and most importantly, most previous studies were based on clinical diagnoses of mental disorders derived from registries or hospital health records. However, we used self-report measures of depressive and anxiety symptoms with data collected independently of hospitals or any other health services. Thereby we reduced the risk of collider bias or selection bias, also known as Berkson's bias. Collider bias may lead to spurious associations in the context of research on COVID-19 risk factors (Griffith *et al*., 2020). However, our sample did not consist of a representative population sample tested for active infection independent of symptomatology. More precisely, if a person was tested or not dependent on government testing guidelines at that time. Hence, we cannot exclude that factors linked to testing influenced our results. Second, the self-report diagnostic measures that we used are well established with good sensitivity and specificity to detect clinically-relevant depression and anxiety (Spitzer, 1999; Spitzer *et al*., 2000, 2006; Kroenke *et al*., 2001; Kroenke and Spitzer, 2002; Löwe *et al*., 2008; Dear *et al*., 2011). This contrasts with previous studies that relied on information on mental disorders based on registry or health records. The latter are related to substantial under-diagnosing of mood and anxiety disorders (Cornelius *et al*., 2014). However, using PHQ-9 comes with a risk of over-diagnosing the presence of major depressive disorders (Levis *et al*., 2020). Of note, in the present study, we examined the association between depressive symptomatology (as opposed to the diagnosis of a mental disorder) and the risk of being diagnosed with COVID-19. Third, we used assessment tools that allow grading the extent and severity of depression and anxiety and thus to estimate the magnitude and dose–response of clinically relevant depression and anxiety on COVID-19. Hence, we provide evidence for a dose–response relationship, which may suggest causality (Hill, 1965; Howick *et al*., 2009). Fourth, our analyses are based on prospective data, with information on depression and anxiety collected between July 2016 and July 2017, long before the onset of the COVID-19 pandemic. In contrast to cross-sectional studies, we provide strong evidence for temporarity, excluding the possibility of COVID-19 increasing the risk of depressive symptoms (Bo *et al*., 2020; Cao *et al*., 2020; Kong *et al*., 2020; Krishnamoorthy *et al*., 2020; Li *et al*., 2020*c*; Mazza *et al*., 2020; Rogers *et al*., 2020). Fifth, we adjusted our estimates for several potential confounders, including physical diseases, assessed before the start of the COVID-19 pandemic, thereby reducing the risk of residual confounding.

Our study has several limitations. First, testing for SARS-CoV-2 was clinically and not study driven. Hence, it is difficult to untangle other factors that may affect the likelihood of being tested for COVID-19. In addition, our study might underestimate negative SARS-CoV-2 test results because not all laboratories reported these (UK Biobank, 2020). Nevertheless, the majority of laboratories reported positive as well as negative SARS-CoV-2 test results. Second, depressive and anxiety symptoms were assessed between July 2016 and July 2017, two and a half to three and a half years before the beginning of the COVID-19 pandemic. Notably, it is rather common for depressive symptoms to be stable over a period of time, particularly among adults (Musliner *et al*., 2016). However, there is substantial heterogeneity in temporal depressive symptom patterns assessed with the PHQ-9, with a subgroup of subjects suffering from depression characterised by fluctuating symptom intensity (Patten and Schopflocher, 2009). Hence, we cannot exclude that some subjects may have recovered from clinically relevant depressive symptoms between assessment and onset of the COVID-19 pandemic, and others may have had a new onset.

Third, the vast majority of subjects tested for COVID-19 in the UK between March and May 2020 presented with severe symptoms, and were suspected of having severe disease courses. Therefore, a positive COVID-19 test in our sample may be biased by severe COVID-19 cases (Armstrong *et al*., 2020). Our analysis may not have captured milder or asymptomatic cases of COVID-19 in the UK Biobank population. Notably, if persons with depressive symptoms and SARS-CoV-2 infection, as compared to persons without depressive symptoms but SARS-CoV-2 infection, are more likely to get tested for COVID-19, this could lead to bias away from the null with regard to an association between depressive symptoms and receiving a diagnosis of COVID-19. Even though we calculated additional prediction models in those being tested for SARS-CoV-2, there is still need for future studies to elucidate the role of symptoms of mental disorders in the context of COVID-19, including their link to testing likelihood.

We should exercise caution when it comes to generalising our results. First, the UK Biobank is not representative of the UK population because of the *healthy volunteer bias* (Fry *et al*., 2017). However, it is one of the largest samples providing prospective data to estimate temporal associations between depressive and anxiety symptoms and subsequent COVID-19. Second, we had to exclude all UK Biobank participants outside England, because there was no information on testing for COVID-19 available for them at the time. Third, participants of the UK Biobank were between 37 and 73 years old at recruitment from March 2006 until December 2010 (Collins, 2012; Sudlow *et al*., 2015; Ho *et al*., 2020). Consequently, caution should be exercised in generalising our observations to subjects younger than 49 or older than 82 years of age. However, as the risk of severe or fatal courses of COVID-19 increases with age, we may have captured most of the relevant decades of life in the context of severe COVID-19 outcomes.

Our findings further substantiate the clinical relevance and weight of mental disorders, particularly depression, as a risk factor for a COVID-19 diagnosis beyond risk factors such as obesity, diabetes and cardiovascular conditions. Additionally, our results highlight the need to differentiate between the predictors of being tested for COVID-19 and the predictors of a positive test result when tested for COVID-19. Notably, this has rarely been addressed in previous studies on other risk factors for COVID-19. Future studies elucidating the role of symptoms of pre-existing mental disorders in representative samples randomly screened for COVID-19 are highly warranted. Yet, information on pre-existing symptoms would most likely be collected retrospectively, potentially inducing recall bias. In general, there is a strong need for a better understanding of the role of symptoms of mental disorders in the context of COVID-19, including how these are involved in risk behaviour, viral exposure, immune function, disease progress, symptom perception, health care use and testing likelihood. We feel strongly that depressive symptoms should be identified and addressed at early stages, for example, by incorporating collaborative care approaches (Carlo *et al*., 2020). This could have the potential to mitigate the risk of infection or severe disease courses related to SARS-CoV-2 and other viruses.

## Conclusion

Based on prospective and self-report data on the symptom severity of mental disorders in a large and nationwide sample, we provide evidence that (a) depressive symptoms but not anxiety are linked to an increased likelihood of being tested for SARS-CoV-2 and (b) depressive symptoms are associated with an increased risk of a diagnosis of COVID-19, irrespective of potential confounders. While depressive symptoms but not anxiety were linked to an increased likelihood of being tested for SARS-CoV-2, there was no such association with a COVID-19 diagnosis in those tested. This stresses the need for a better understanding of potential underlying mechanisms, including risk behaviour, viral exposure, immune function, disease progress, symptom perception, health care use and testing likelihood. Our findings highlight the relevance of mental processes in the context of COVID-19.

## Data Availability

We requested and retrieved the data from the UK Biobank in accordance with their guidelines and policies, which do not allow us to transfer data to third parties. Those interested in working with UK Biobank data can find information on how to apply and access UK Biobank on the UK Biobank website (https://www.ukbiobank.ac.uk/enable-your-research).

## Supplementary material

For supplementary material accompanying this paper visit http://doi.org/10.1017/S2045796021000676. click here to view supplementary material
