# Supplementary material for: Depressive symptoms, but not anxiety, predict subsequent diagnosis of Coronavirus disease 19: a national cohort study
Source: Epidemiol Psychiatr Sci. 2022 Mar 25;31:e16. doi: 10.1017/S2045796021000676 (PMC8967696; doi:10.1017/S2045796021000676)
Supplement: Supplementary file 1 [file epssup.zip › S2045796021000676sup001.docx]

# Online Supplemental Material 2: Detailed Statistical Analyses

We performed all calculations at sciCORE (‘SciCORE | Center for Scientific Computing’, n.d.) scientific computing centre at the University of Basel. We provided descriptive information on median and interquartile range for non-normally distributed continuous variables and absolute and relative frequencies for categorical variables.

To estimate the association of depressive and anxiety symptoms with COVID-19, we conducted logistic regression analyses. For the crude models, we entered depressive and anxiety symptom scores as continuous predictor variables with COVID-19 as the main outcome. For adjusted models, we conducted a two-step adjustment scheme. As a first step, we concomitantly adjusted the analyses for *a priori* defined sociodemographic variables, acting as potential confounders: age, sex, ethnicity, and deprivation index, categorised as outlined in Table 1 in the main article. As a second step, we adjusted analyses for *a priori* selected physical diseases and behavioural risk factors (see Table 1 in the main article) that have been reported elsewhere as being linked to an increased risk of COVID-19 (Zhou et al., 2020; Wang et al., 2020). These may potentially act as confounders or mediators, given the lack of information on the timing of these physical diseases and behavioural risk factors as compared to the depressive and anxiety symptoms. For the second adjustment step, we entered first step covariates, as well as one physical disease or behavioural risk factor at a time.

Next, to estimate the association between depressive and anxiety symptoms with *being tested for SARS-CoV-2*, we conducted additional logistic regression analyses. Here again, we applied the two step adjustment scheme outlined above.

Further, to estimate the association of depressive and anxiety symptoms with COVID-19 *in those being tested for SARS-CoV-2*, we conducted logistic regression analyses as outlined above, this time however restricting the analyses to participants who had been tested for SARS-CoV-2. To prevent overfitting, we adjusted for only one sociodemographic variable at a time in step 1 and omitted adjusting for step 1 covariates during step 2.

We excluded participants who did not answer all the questions in the mental health web-based questionnaire necessary to calculate GAD-7 and PHQ-9 scores. We handled missing data by conducting completer analyses, excluding participants who lacked information on ethnicity, Townsend score, BMI, or smoking and drinking status, when adjusting for these confounders, respectively (see Tables 4–8 in online supplemental material 3).

We tested the linearity assumption between continuous predictor variables and the logit (log of odds ratio) of the outcome. For this, we inspected the scatter plot between each predictor and the logit values. We tested for collinearity between the continuous predictors GAD-7 and PHQ-9 scores by calculating the variance inflation factors (VIF).

We provided estimates with 95% confidence intervals, in accordance with two-tailed p-values with a statistical significance level of 0.05.

We used the statistical software package R version 4.0.0 and above (R Core Team, 2020) for all data analyses, visualisation and statistical testing. In addition to base R, we used the following R packages: for data preparation and descriptive statistics "dplyr" (Wickham et al., 2020), "tidyr" (Wickham & Henry, 2020), "forcats" (Wickham, 2020), "broom" (Robinson & Hayes 2020), "lubridate" (Grolemund & Wickham, 2011); for VIF calculations "car" (Fox & Weisberg, 2019); for data visualisation "ggplot2" (Wickham, 2016).

References of Online Supplemental Material 2

**Fox J, Weisberg S** (2019) *An R Companion to Applied Regression*, 3rd ed. Thousand Oaks CA, Sage Publications (https://socialsciences.mcmaster.ca/jfox/Books/Companion/).

**Grolemund G, Wickham H** (2011) Dates and Times Made Easy with lubridate. *Journal of Statistical Software*, 40(3). (http://www.jstatsoft.org/v40/i03/). Accessed 6 October 2020.

**R Core Team** (2020) *R: A Language and Environment for Statistical Computing*. Vienna, Austria (https://www.R-project.org/).

**Robinson D, Hayes A** (2020) *A. broom: Convert Statistical Analysis Objects into Tidy Tiblles. R package version 0.5.6. 2020*. (https://CRAN.R-project.org/package=broom).

**sciCORE | Center for Scientific Computing** (n.d.) [web site]. (https://scicore.unibas.ch). Accessed 6 October 2020.

**Wang B, Li R, Lu Z, Huang Y** (2020) Does comorbidity increase the risk of patients with COVID-19: evidence from meta-analysis. *Aging*, 12(7):6049–6057. (https://www.aging-us.com/lookup/doi/10.18632/aging.103000). Accessed 27 August 2020.

**Wickham H** (2016) *ggplot2: Elegant Graphics for Data Analysis*. New York, Springer-Verlag (https://ggplot2.tidyverse.org/).

**Wickham H** (2020) *forcats: Tools for Working with Categorical Variables (Factors). R package version 0.5.0. 2020.* (https://CRAN.R-project.org/package=forcats).

**Wickham H, François R, Henry L, Müller K** (2020) *dplyr: A Grammar of Data Manipulation. R package version 0.8.5. 2020*. (https://dplyr.tidyverse.org).

**Wickham H, Henry L** (2020) *tidyr: Tidy Messy Data. R package. R package version 1.0.2. 2020*. (https://CRAN.R-project.org/package=tidyr).

**Zhou Y, Yang Q, Chi J, Dong B, Lv W, Shen L, Wang Y** (2020) Comorbidities and the risk of severe or fatal outcomes associated with coronavirus disease 2019: A systematic review and meta-analysis. *International Journal of Infectious Diseases*, 99:47–56. (https://linkinghub.elsevier.com/retrieve/pii/S1201971220305725). Accessed 27 August 2020.
