# Supplementary material for: Depressive symptoms, but not anxiety, predict subsequent diagnosis of Coronavirus disease 19: a national cohort study
Source: Epidemiol Psychiatr Sci. 2022 Mar 25;31:e16. doi: 10.1017/S2045796021000676 (PMC8967696; doi:10.1017/S2045796021000676)
Supplement: Supplementary file 1 [file epssup.zip › S2045796021000676sup002.docx]

# Online Supplemental Material 3: Detailed Results

Relative frequencies of COVID-19 and being tested for SARS-CoV-2 in our sample stratified for depressive and anxiety symptom severity categories, age, and sex are provided in Online Supplemental Tables 1–3. Of note, 502 participants in our sample died between January 2^nd^ 2020 and August 24^th^ 2020, of whom 53 had a COVID-19 related cause of death recorded in the death register (ICD-10 codes U071 and U072).

Online Supplemental Tables 4–8 detail the odds ratios (*OR*), 95% confidence intervals (CI) and *p*-values when adjusting for potential sociodemographic confounders, individual physical diseases and behavioural factors.

Regarding the assumptions of logistic regression analyses, visual inspection of the smoothed out scatter plot of the continuous predictors PHQ-9 and GAD-7 scores versus the logit of the outcomes indicated strong linearity across the range of PHQ-9 and GAD-7. There was some uncertainty about linearity in the upper range of PHQ-9 and GAD-7 in the tested sample, given the limited number of positive COVID-19 cases in those ranges. All VIF values were smaller than 2.2, indicating no risk of relevant multicollinearity (Hair et al., 2010).

**Online Supplemental Table 1. SARS-CoV-2 Tests and COVID-19 Stratified for Depressive Symptom Severity Categories**

| **PHQ-9 Category** | **Score range, meaning** | **Frequency** |  | **Tested for SARS-CoV-2** | |  | **Confirmed COVID-19** | | |
| --- | --- | --- | --- | --- | --- | --- | --- | --- | --- |
|  |  |  |  | **Frequency tested** | **Percentage** |  | **Frequency** | **Percentage with respect to tested** | **Percentage with respect to total** |
| 0 | 0–4, minimal | 107,772 |  | 3,659 | 3.40% |  | 249 | 6.81% | 0.23% |
| 1 | 5–9, mild | 19,702 |  | 773 | 3.92% |  | 66 | 8.54% | 0.33% |
| 2 | 10–14, moderate | 4,989 |  | 252 | 5.05% |  | 22 | 8.73% | 0.44% |
| 3 | 15–27, (moderately) severe | 2,639 |  | 143 | 5.42% |  | 13 | 9.09% | 0.49% |
| **Totals** |  | **135,102** |  | **4,827** | **3.57%** |  | **350** | **7.25%** | **0.26%** |

Abbreviations: COVID-19, Coronavirus disease 19; SARS-CoV-2, Severe acute respiratory syndrome coronavirus 2.

**Online Supplemental Table 2. SARS-CoV-2 Tests and COVID-19 Stratified for Anxiety Symptom Severity Categories**

| **GAD-7 Category** | **Score range, meaning** | **Frequency** |  | **Tested for SARS-CoV-2** | |  | **Confirmed COVID-19** | | |
| --- | --- | --- | --- | --- | --- | --- | --- | --- | --- |
|  |  |  |  | **Frequency tested** | **Percentage tested** |  | **Frequency** | **Percentage with respect to tested** | **Percentage with respect to total** |
| 0 | 0–4, none | 111,024 |  | 3,846 | 3.46% |  | 264 | 6.86% | 0.24% |
| 1 | 5–9, mild | 18,381 |  | 727 | 3.96% |  | 59 | 8.12% | 0.32% |
| 2 | 10–14, moderate | 3,663 |  | 164 | 4.48% |  | 16 | 9.76% | 0.44% |
| 3 | 15–21, severe | 2,034 |  | 90 | 4.42% |  | 11 | 12.22% | 0.54% |
| **Totals** |  | **135,102** |  | **4,827** | **3.57%** |  | **350** | **7.25%** | **0.26%** |

Abbreviations: COVID-19, Coronavirus disease 19; SARS-CoV-2, Severe acute respiratory syndrome coronavirus 2.

**Online Supplemental Table 3. SARS-CoV-2 Tests and COVID-19 Stratified for Age Intervals and Sex**

| **Age intervals (years)** | **Absolute frequency / subjects (%)** | | | | |
| --- | --- | --- | --- | --- | --- |
|  | **Tested for SARS-CoV-2** | |  | **Confirmed COVID-19** | |
|  | **Female** | **Male** |  | **Female** | **Male** |
| 49–55 | 252/6,682 (3.8%) | 126/4,631 (2.7%) |  | 38/6,682 (0.6%) | 21/4,631 (0.5%) |
| 55–59 | 394/10,808 (3.6%) | 198/7,050 (2.8%) |  | 52/10,808 (0.5%) | 31/7,050 (0.4%) |
| 60–64 | 400/13,439 (3.0%) | 232/8,534 (2.7%) |  | 35/13,439 (0.3%) | 20/8,534 (0.2%) |
| 65–69 | 457/15,737 (2.9%) | 357/11,433 (3.1%) |  | 23/15,737 (0.1%) | 18/11,433 (0.2%) |
| 70–74 | 613/18,266 (3.4%) | 645/15,323 (4.2%) |  | 22/18,266 (0.1%) | 33/15,323 (0.2%) |
| 75–79 | 427/10,310 (4.1%) | 569/10,384 (5.5%) |  | 13/10,310 (0.1%) | 37/10,384 (0.4%) |
| 80–83 | 64/1,215 (5.3%) | 93/1,290 (7.2%) |  | 2/1,215 (0.2%) | 5/1,290 (0.4%) |
| **Total** | 2,607/76,457 (3.4%) | 2,220/58,645 (3.8%) |  | 185/76,457 (0.2%) | 165/58,645 (0.3%) |

Abbreviations: COVID-19, Coronavirus disease 19; SARS-CoV-2, Severe acute respiratory syndrome coronavirus 2.

**Online Supplemental Table 4. Depressive and Anxiety Symptoms Predicting COVID-19 in Total Sample, Adjusted for Sociodemographic Factors**

|  | ***OR* [95% CI]** | ***p*-value** |
| --- | --- | --- |
| PHQ-9 score | 1.037 [1.002, 1.072] | 0.034 |
| GAD-7 score | 1.005 [0.965, 1.044] | 0.82 |
| Sex female | Reference category |  |
| Sex male | 1.225 [0.99, 1.515] | 0.061 |
| Age^a^ 49–55 | Reference category |  |
| Age 55–59 | 0.911 [0.652, 1.28] | 0.59 |
| Age 60–64 | 0.521 [0.359, 0.754] | 0.00054 |
| Age 65–69 | 0.33 [0.219, 0.493] | <0.0001 |
| Age 70–74 | 0.368 [0.252, 0.536] | <0.0001 |
| Age 75–79 | 0.546 [0.37, 0.803] | 0.0021 |
| Age 80–83 | 0.638 [0.264, 1.311] | 0.26 |
| Ethnicity^b^ white | Reference category |  |
| Ethnicity black | 3.159 [1.652, 5.476] | 0.00015 |
| Ethnicity south Asian | 1.803 [0.765, 3.551] | 0.13 |
| Ethnicity other | 1.534 [0.727, 2.822] | 0.21 |
| Townsend^b^ category least deprived | Reference category |  |
| Townsend category average | 1.251 [0.985, 1.584] | 0.064 |
| Townsend category most deprived | 1.447 [1.066, 1.94] | 0.015 |

Abbreviations: CI, confidence interval; Coronavirus disease 19, COVID-19; OR, odds ratio.

^a^ Age in years.

^b^ Excluded 585 participants with no information on ethnicity or Townsend score.

**Online Supplemental Table 5. Depressive and Anxiety Symptoms Predicting COVID-19 in Total Sample, Adjusted for Individual Physical Diseases and Behavioural Factors**

|  | **Depressive symptoms** |  | **Anxiety symptoms** |  |
| --- | --- | --- | --- | --- |
| **Adjusted for:** | ***OR* [95% CI]** | ***p*-value** | ***OR* [95% CI]** | ***p*-value** |
| Asthma | 1.05 [1.015, 1.084] | 0.0036 | 1.009 [0.97, 1.047] | 0.65 |
| Cancer | 1.052 [1.017, 1.086] | 0.0024 | 1.009 [0.97, 1.047] | 0.65 |
| Cerebrovascular disease | 1.051 [1.016, 1.085] | 0.003 | 1.009 [0.971, 1.048] | 0.63 |
| COPD | 1.05 [1.015, 1.084] | 0.0035 | 1.009 [0.97, 1.048] | 0.64 |
| Coronary artery disease | 1.048 [1.014, 1.083] | 0.0045 | 1.011 [0.972, 1.049] | 0.59 |
| Diabetes mellitus | 1.047 [1.012, 1.082] | 0.0056 | 1.011 [0.972, 1.05] | 0.57 |
| Hypertension | 1.048 [1.013, 1.082] | 0.005 | 1.011 [0.972, 1.05] | 0.57 |
| Obesity^a^ | 1.038 [1.003, 1.073] | 0.029 | 1.015 [0.976, 1.054] | 0.44 |
| Morbid obesity^a^ | 1.04 [1.005, 1.075] | 0.022 | 1.013 [0.974, 1.052] | 0.5 |
| Lifetime smoking^b^ | 1.051 [1.016, 1.085] | 0.0029 | 1.009 [0.97, 1.048] | 0.64 |
| Lifetime drinking^c^ | 1.051 [1.017, 1.086] | 0.0025 | 1.009 [0.97, 1.047] | 0.65 |

Abbreviations: CI, confidence interval; COVID-19, Coronavirus disease 19; OR, odds ratio.

^a^ Excluded 283 participants with unknown body mass index.

^b^ Excluded 266 participants with unknown smoking status.

^c^ Excluded 107 participants with unknown drinking status.

**Online Supplemental Table 6. Depressive and Anxiety Symptoms Predicting Getting Tested for SARS-CoV-2, Adjusted for Sociodemographic Factors**

|  | ***OR* [95% CI]** | ***p*-value** |
| --- | --- | --- |
| PHQ-9 score | 1.042 [1.032, 1.053] | <0.0001 |
| GAD-7 score | 0.993 [0.981, 1.005] | 0.24 |
| Sex female | Reference category |  |
| Sex male | 1.104 [1.041, 1.171] | 0.00087 |
| Age^a^ 49–55 | Reference category |  |
| Age 55–59 | 1.008 [0.883, 1.151] | 0.91 |
| Age 60–64 | 0.902 [0.792, 1.029] | 0.12 |
| Age 65–69 | 0.971 [0.857, 1.102] | 0.65 |
| Age 70–74 | 1.243 [1.104, 1.402] | 0.00035 |
| Age 75–79 | 1.625 [1.437, 1.84] | <0.0001 |
| Age 80–83 | 2.156 [1.773, 2.61] | <0.0001 |
| Ethnicity^b^ white | Reference category |  |
| Ethnicity black | 1.253 [0.915, 1.672] | 0.14 |
| Ethnicity south Asian | 1.187 [0.88, 1.563] | 0.24 |
| Ethnicity other | 1.296 [1.026, 1.612] | 0.024 |
| Townsend^b^ category least deprived | Reference category |  |
| Townsend category average | 1.069 [1.001, 1.141] | 0.045 |
| Townsend category most deprived | 1.302 [1.194, 1.418] | <0.0001 |

Abbreviations: CI, confidence interval; OR, odds ratio; SARS-CoV-2, Severe acute respiratory syndrome coronavirus 2.

^a^ Age in years.

^b^ Excluded 585 participants with no information on ethnicity or Townsend score.

**Online Supplemental Table 7. Depressive and Anxiety Symptoms Predicting Getting Tested for SARS-CoV-2, Adjusted for Sociodemographic Factors, Individual Physical Diseases, and Behavioural Factors**

| **Adjusted for sociodemographic^a^ factors and:** | **Depressive symptoms** |  | **Anxiety symptoms** |  |
| --- | --- | --- | --- | --- |
|  | ***OR* [95% CI]** | ***p*-value** | ***OR* [95% CI]** | ***p*-value** |
| Asthma | 1.041 [1.031, 1.052] | <0.0001 | 0.993 [0.981, 1.005] | 0.24 |
| Cancer | 1.042 [1.031, 1.053] | <0.0001 | 0.993 [0.981, 1.005] | 0.24 |
| Cerebrovascular disease | 1.041 [1.031, 1.052] | <0.0001 | 0.994 [0.982, 1.005] | 0.26 |
| COPD | 1.04 [1.029, 1.051] | <0.0001 | 0.993 [0.981, 1.005] | 0.24 |
| Coronary artery disease | 1.04 [1.029, 1.05] | <0.0001 | 0.994 [0.982, 1.005] | 0.27 |
| Diabetes mellitus | 1.039 [1.028, 1.05] | <0.0001 | 0.994 [0.983, 1.006] | 0.34 |
| Hypertension | 1.036 [1.026, 1.047] | <0.0001 | 0.995 [0.983, 1.006] | 0.35 |
| Obesity^b^ | 1.036 [1.025, 1.047] | <0.0001 | 0.996 [0.985, 1.008] | 0.53 |
| Morbid obesity^b^ | 1.037 [1.026, 1.048] | <0.0001 | 0.996 [0.984, 1.008] | 0.49 |
| Lifetime smoking^c^ | 1.042 [1.031, 1.052] | <0.0001 | 0.993 [0.981, 1.005] | 0.21 |
| Lifetime drinking^d^ | 1.042 [1.031, 1.053] | <0.0001 | 0.993 [0.981, 1.005] | 0.24 |

Abbreviations: CI, confidence interval; COPD, chronic obstructive pulmonary disease; COVID-19, Coronavirus disease 19; OR, odds ratio; SARS-CoV-2, Severe acute respiratory syndrome coronavirus 2.

^a^ Excluded 585 participants with no information on ethnicity or Townsend score.

^b^ Excluded 854 participants with no information on ethnicity, Townsend score or body mass index.

^c^ Excluded 787 with no information on ethnicity, Townsend score or smoking status.

^d^ Excluded 631 with no information on ethnicity, Townsend score or drinking status.

**Online Supplemental Table 8. Depressive and Anxiety Symptoms Predicting COVID-19 Among Tested Subjects, Adjusted for Individual Sociodemographic, Individual Physical Diseases and Behavioural Factors**

|  | **Depressive symptoms** |  | **Anxiety symptoms** |  |
| --- | --- | --- | --- | --- |
| **Adjusted for:** | ***OR* [95% CI]** | ***p*-value** | ***OR* [95% CI]** | ***p*-value** |
| Sex | 1.015 [0.981, 1.05] | 0.38 | 1.022 [0.981, 1.062] | 0.28 |
| Age category | 1.006 [0.972, 1.041] | 0.71 | 1.009 [0.968, 1.05] | 0.67 |
| Ethnicity^a^ | 1.016 [0.981, 1.051] | 0.35 | 1.015 [0.974, 1.056] | 0.47 |
| Townsend category^b^ | 1.012 [0.978, 1.046] | 0.49 | 1.022 [0.982, 1.063] | 0.27 |
| Asthma | 1.014 [0.979, 1.048] | 0.42 | 1.021 [0.981, 1.061] | 0.3 |
| Cancer | 1.015 [0.981, 1.049] | 0.38 | 1.021 [0.98, 1.061] | 0.31 |
| Cerebrovascular disease | 1.015 [0.981, 1.05] | 0.38 | 1.021 [0.981, 1.061] | 0.3 |
| COPD | 1.015 [0.981, 1.05] | 0.37 | 1.021 [0.981, 1.061] | 0.3 |
| Coronary artery disease | 1.014 [0.98, 1.049] | 0.41 | 1.022 [0.981, 1.062] | 0.28 |
| Diabetes mellitus | 1.014 [0.98, 1.049] | 0.4 | 1.021 [0.981, 1.062] | 0.29 |
| Hypertension | 1.017 [0.982, 1.051] | 0.33 | 1.02 [0.98, 1.061] | 0.32 |
| Obesity^c^ | 1.007 [0.972, 1.042] | 0.7 | 1.024 [0.983, 1.065] | 0.24 |
| Morbid obesity^c^ | 1.009 [0.974, 1.044] | 0.62 | 1.023 [0.982, 1.063] | 0.26 |
| Lifetime smoking^d^ | 1.015 [0.981, 1.049] | 0.38 | 1.021 [0.981, 1.062] | 0.3 |
| Lifetime drinking^e^ | 1.015 [0.981, 1.05] | 0.38 | 1.021 [0.981, 1.062] | 0.3 |

Abbreviations: CI, confidence interval; COPD, chronic obstructive pulmonary disease; COVID-19, Coronavirus disease 19; OR, odds ratio.

^a^ Excluded 11 participants missing information on ethnicity.

^b^ Excluded 7 participants with unknown Townsend score.

^c^ Excluded 19 participants with unknown body mass index.

^d^ Excluded 7 participants with unknown smoking status..

^e^ Excluded 1 participant with unknown drinking status.

References of Online Supplemental Material 3

**Hair J, Black W, Babin B, Anderson R** (2010) *Multivariate data analysis*, 7th ed. Upper saddle River, New Jersey, Pearson Education International.
