# Supplementary material for: Depressive symptoms, but not anxiety, predict subsequent diagnosis of Coronavirus disease 19: a national cohort study
Source: Epidemiol Psychiatr Sci. 2022 Mar 25;31:e16. doi: 10.1017/S2045796021000676 (PMC8967696; doi:10.1017/S2045796021000676)
Supplement: Supplementary file 1 [file epssup.zip › S2045796021000676sup003.docx]

# Online Supplemental Material 1: Detailed Study Methods

### Outline of the Study Cohort

The present study is based on the UK Biobank, a large population-based national cohort of United Kingdom (UK) residents (Smith et al., 2013). Between March 2006 and December 2010, 502,624 voluntary participants aged between 37 and 73 years were recruited for the UK Biobank. The recruitment took place in 22 assessment centres across England, Scotland, and Wales (Collins, 2012; Sudlow et al., 2015; Fry et al., 2017; ‘About UK Biobank’, 2020; Ho et al., 2020).

At baseline assessment, participants provided information on sociodemographic characteristics and self-reported health, amongst other information. Over the years, subsamples of UK Biobank participants replied to various online self-reported questionnaires on selected topics, including a mental health web-based questionnaire from July 2016 to July 2017 (UK Biobank, 2017; Davis et al., 2020;). Additionally, a proportion of participants were invited to a repeat assessment, an imaging assessment and a repeat imaging assessment in 2012, 2014 and 2019 respectively. During these subsequent visits, some information that was missing at the initial assessment was collected. Further, information from the hospital inpatient data was linked to the UK Biobank dataset (UK Biobank, 2020b), cancer register (UK Biobank, 2013), death register (UK Biobank, 2020a), and primary care data (UK Biobank, 2019). Additional information on assessments conducted with the UK Biobank participants is provided elsewhere (Sudlow et al., 2015; ‘About UK Biobank’, 2020). Public Health England (PHE) provided information on testing for SARS-CoV-2 in the UK Biobank participants (Armstrong et al., 2020).

All participants provided written informed consent on touchscreen at baseline assessment. The UK Biobank received ethical approval from the North West Multi-Centre Research Ethics Committee (REC reference: 11/NW/03820) (Collins, 2012; Sudlow et al., 2015). Analyses for this publication were performed under UK Biobank application number 49658.

### Collected Information

#### Sociodemographic Characteristics

We used the month and year of birth from the National Health Service (NHS) central registry to calculate the age of the study participants on January 1^st^ 2020. To ensure privacy, no day of birth was provided so we arbitrarily chose the 15th as the day of birth. We categorised the participants’ ages on January 1^st^ 2020 into seven categories, as outlined in Table 1 in the main article. Information on sex was acquired from the NHS central registry and was at times updated by participants. Therefore, information on sex can differ from information on sex as biologically determined at birth. Information on ethnicity was collected at baseline assessment by self-report according to prespecified categories. We categorised ethnicity as white, black, south Asian, and other (see Table 1 in the main article). Participants were assigned a Townsend deprivation score (Fry et al., 2017) corresponding to the output area of their residential postcode at baseline assessment (categorised as least deprived: < -2.00; average: -2.00 to 1.99; most deprived: ≥ 2.00).

#### Physical Diseases and Behavioural Risk Factors

Data for asthma (Zhang et al., 2018a), cancer, cerebrovascular disease, chronic obstructive pulmonary disease (COPD), coronary artery disease, diabetes mellitus, and hypertension were generated by mapping primary care data from general practitioners, hospital inpatient data, death register records, and self-reported at verbal interviews and touchscreen questionnaires at baseline or subsequent UK Biobank assessment centre visits. We considered a physical disease present if any of these sources indicated its occurrence at least once in the participant's lifetime.

Cerebrovascular disease included stroke, subarachnoid haemorrhage, brain haemorrhage, and ischaemic stroke. COPD includes emphysema and chronic bronchitis (Zhang et al., 2018b). Coronary artery disease included myocardial infarction and angina pectoris. Diabetes mellitus included insulin-dependent, non–insulin-dependent, malnutrition-related, other specified and unspecified diabetes mellitus. Hypertension included primary and secondary hypertension. Body mass index (BMI) was calculated using height and weight measurements collected at baseline assessment or subsequent UK Biobank assessment centre visits. We classified participants with BMI ≥ 30 kg/m^2^ as obese and BMI ≥ 35 kg/m^2^ as morbidly obese. Participants reported their smoking and drinking statuses via a touchscreen questionnaire at baseline assessment or subsequent UK Biobank assessment centre visits (UK Biobank, n.d.-a). We used the most recent self-reported information on BMI, smoking and drinking statuses. We only labelled participants as lifetime non-smokers or non-drinkers if they reported not smoking, respectively not drinking, at all assessment visits when the information was available. We recorded information on cause of death from the death register linked to the UK Biobank; however, we did not estimate the association of depression and anxiety with COVID-19 related death, given the low number of cases in our UK biobank sample.

#### Depressive and Anxiety Symptoms

The UK Biobank used the 9-item Patient Health Questionnaire Scale (PHQ-9, cronbach's α = 0.89, test-retest reliability *r* = 0.84) to assess depressive symptoms ( Spitzer, 1999; Spitzer et al., 2000; Kroenke et al., 2001, 2002) and the 7-item generalised Anxiety Disorder Scale (GAD-7, cronbach's α = 0.89–0.92, test–retest reliability *r* = 0.83) to assess anxiety symptoms ( Spitzer et al., 2006; Löwe et al., 2008; Dear et al., 2011). We computed PHQ-9 and GAD-7 scores as previously defined (Kroenke & Spitzer, 2002; Spitzer et al., 2006). For most analyses and if not explicitly mentioned, we entered them as continuous variables. Otherwise, we applied previously published severity category cut-offs (Kroenke et al., 2010), merging moderately severe and severe categories as outlined in Figure 2 in the main article.

#### Testing for SARS-CoV-2 and COVID-19 Case Definition

Testing for SARS-CoV-2 in England was conducted in accident and emergency departments and in hospital settings. These data were provided by PHE and linked through the SGSS database to the UK Biobank data. Data on SARS-CoV-2 polymerase chain reaction (PCR) tests provided by PHE included the specimen date, specimen type (e.g. nasal, sputum etc.), laboratory, origin (evidence from microbiological record that the participant was an inpatient or not) and result (positive or negative). Data were available for the period March 16^th^ 2020 to August 24^th^ 2020. Testing was conducted in 128 laboratories across England (Armstrong et al., 2020; UK Biobank, n.d.-b). We considered a UK Biobank participant as being tested if they had at least one test result (positive or negative) in the SGSS database.

We defined a confirmed case of COVID-19 as meeting confirmatory laboratory evidence by detecting “severe acute respiratory syndrome coronavirus 2 ribonucleic acid” (SARS-CoV-2 RNA) in at least one clinical specimen provided by the index subject, using a molecular amplification detection test. This case definition is in line with i) the “Coronavirus Disease 2019 (COVID-19) 2020 Interim Case Definition” by the United States *Centre for Disease Control and Prevention* (‘Coronavirus Disease 2019 (COVID-19) | 2020 Interim Case Definition, Approved April 5, 2020’, 2020) and ii) the case definition for coronavirus disease 2019 as of 29 May 2020 by the *European Centre for Disease and Control* (‘Case Definition for Coronavirus Disease 2019 (COVID-19), as of 29 May 2020’, 2020)*.*

## References of Online Supplemental Material 1

**About UK Biobank** (2020) [web site]. (<https://www.ukbiobank.ac.uk/about-biobank-uk/>). Accessed 21 September 2020.

**Armstrong J, Rudkin JK, Allen N, Crook DW, Wilson DJ, Wyllie DH, O’Connell AM** (2020) Dynamic linkage of COVID-19 test results between Public Health England’s Second Generation Surveillance System and UK Biobank. *Microbial Genomics*. (<https://www.microbiologyresearch.org/content/journal/mgen/10.1099/mgen.0.000397>). Accessed 28 July 2020.

**Case definition for coronavirus disease 2019 (COVID-19), as of 29 May 2020** (2020) [web site]. (https://www.ecdc.europa.eu/en/covid-19/surveillance/case-definition). Accessed 25 August 2020.

**Collins R** (2012) What makes UK Biobank special? *The Lancet*, 379(9822):1173–1174. (<https://linkinghub.elsevier.com/retrieve/pii/S0140673612604048>). Accessed 22 October 2020.

**Coronavirus Disease 2019 (COVID-19) | 2020 Interim Case Definition, Approved April 5, 2020** (2020) [web site]. (https://wwwn.cdc.gov/nndss/conditions/coronavirus-disease-2019-covid-19/case-definition/2020/). Accessed 25 August 2020.

**Davis KAS, Coleman JRI, Adams M, Allen N, Breen G, Cullen B, Dickens C, Fox E, Graham N, Holliday J, Howard LM, John A, Lee W, McCabe R, McIntosh A, Pearsall R, Smith DJ, Sudlow C, Ward J, Zammit S, Hotopf M** (2020) Mental health in UK Biobank – development, implementation and results from an online questionnaire completed by 157 366 participants: a reanalysis. *BJPsych Open*, 6(2):e18. (https://www.cambridge.org/core/product/identifier/S2056472419001005/type/journal_article). Accessed 30 April 2020.

**Dear BF, Titov N, Sunderland M, McMillan D, Anderson T, Lorian C, Robinson E** (2011) Psychometric Comparison of the Generalized Anxiety Disorder Scale-7 and the Penn State Worry Questionnaire for Measuring Response during Treatment of Generalised Anxiety Disorder. *Cognitive Behaviour Therapy*, 40(3):216–227. (http://www.tandfonline.com/doi/abs/10.1080/16506073.2011.582138). Accessed 6 October 2020).

**Fry A, Littlejohns TJ, Sudlow C, Doherty N, Adamska L, Sprosen T, Collins R, Allen NE** (2017) Comparison of Sociodemographic and Health-Related Characteristics of UK Biobank Participants With Those of the General Population. *American Journal of Epidemiology*, 186(9):1026–1034. (https://academic.oup.com/aje/article/186/9/1026/3883629). Accessed 20 April 2020).

**Ho FK, Celis-Morales CA, Gray SR, Katikireddi SV, Niedzwiedz CL, Hastie C, Lyall DM, Ferguson LD, Berry C, Mackay DF, Gill JMR, Pell JP, Sattar N, Welsh PI** (2020) *Modifiable and non-modifiable risk factors for COVID-19: results from UK Biobank*. Epidemiology (http://medrxiv.org/lookup/doi/10.1101/2020.04.28.20083295). Accessed 7 May 2020.

**Kroenke K, Spitzer RL (2002)** The PHQ-9: A New Depression Diagnostic and Severity Measure. *Psychiatric Annals*, 32(9):509–515. (http://www.healio.com/doiresolver?doi=10.3928/0048-5713-20020901-06). Accessed 6 October 2020.

**Kroenke K, Spitzer RL, Williams JBW** (2001) The PHQ-9: Validity of a brief depression severity measure. *Journal of General Internal Medicine*, 16(9):606–613. (http://link.springer.com/10.1046/j.1525-1497.2001.016009606.x). Accessed 6 October 2020.

**Kroenke K, Spitzer RL, Williams JBW, Löwe B** (2010) The Patient Health Questionnaire Somatic, Anxiety, and Depressive Symptom Scales: a systematic review. *General Hospital Psychiatry*, 32(4):345–359. (https://linkinghub.elsevier.com/retrieve/pii/S0163834310000563). Accessed 4 November 2020.

**Löwe B, Decker O, Müller S, Brähler E, Schellberg D, Herzog W, Herzberg PY** (2008) Validation and Standardization of the Generalized Anxiety Disorder Screener (GAD-7) in the General Population: *Medical Care*, 46(3):266–274. (http://journals.lww.com/00005650-200803000-00006). Accessed 11 May 2020.

**Smith DJ, Nicholl BI, Cullen B, Martin D, Ul-Haq Z, Evans J, Gill JMR, Roberts B, Gallacher J, Mackay D, Hotopf M, Deary I, Craddock N, Pell JP** (2013) Prevalence and Characteristics of Probable Major Depression and Bipolar Disorder within UK Biobank: Cross-Sectional Study of 172,751 ParticipantsPotash JB, ed. *PLoS ONE*, 8(11):e75362. (http://dx.plos.org/10.1371/journal.pone.0075362). Accessed 28 April 2020.

**Spitzer RL** (1999) Validation and Utility of a Self-report Version of PRIME-MDThe PHQ Primary Care Study. *JAMA*, 282(18):1737. (http://jama.jamanetwork.com/article.aspx?doi=10.1001/jama.282.18.1737). Accessed 6 October 2020.

**Spitzer RL, Kroenke K, Williams JBW, Löwe B** (2006) A Brief Measure for Assessing Generalized Anxiety Disorder: The GAD-7. *Archives of Internal Medicine*, 166(10):1092. (http://archinte.jamanetwork.com/article.aspx?doi=10.1001/archinte.166.10.1092). Accessed 6 October 2020.

**Spitzer RL, Williams JBW, Kroenke K, Hornyak R, McMurray J** (2000) Validity and utility of the PRIME-MD Patient Health Questionnaire in assessment of 3000 obstetric-gynecologic patients: The PRIME-MD Patient Health Questionnaire Obstetrics-Gynecology Study. *American Journal of Obstetrics and Gynecology*, 183(3):759–769. (https://linkinghub.elsevier.com/retrieve/pii/S0002937800786868). Accessed 6 October 2020.

**Sudlow C, Gallacher J, Allen N, Beral V, Burton P, Danesh J, Downey P, Elliott P, Green J, Landray M, Liu B, Matthews P, Ong G, Pell J, Silman A, Young A, Sprosen T, Peakman T, Collins R** (2015) UK Biobank: An Open Access Resource for Identifying the Causes of a Wide Range of Complex Diseases of Middle and Old Age. *PLOS Medicine*, 12(3):e1001779. (https://dx.plos.org/10.1371/journal.pmed.1001779). Accessed 30 April 2020.

**UK Biobank** (2013) Cancer data: linkage from national cancer registries - Version 1.4. (https://biobank.ndph.ox.ac.uk/showcase/showcase/docs/CancerLinkage.pdf). Accessed 21 September 2020.

**UK Biobank** (2017) Mental health web-based questionnaire - Version 1.3. (http://biobank.ndph.ox.ac.uk/showcase/showcase/docs/mental_health_online.pdf). Accessed 21 September 2020.

**UK Biobank** (2019) Primary Care - Linked Data. (http://biobank.ndph.ox.ac.uk/showcase/showcase/docs/primary_care_data.pdf). Accessed 6 October 2020.

**UK Biobank** (2020a) Mortality data: linkage to death registries. (http://biobank.ndph.ox.ac.uk/showcase/showcase/docs/DeathLinkage.pdf). Accessed 21 September 2020.

**UK Biobank** (2020b) Hospital inpatient data - Version 3.0. (http://biobank.ndph.ox.ac.uk/showcase/showcase/docs/HospitalEpisodeStatistics.pdf). Accessed 21 September 2020.

**UK Biobank** (n.d.-a) UK Biobank touch-screen questionnaire: final version. (https://biobank.ndph.ox.ac.uk/showcase/showcase/docs/TouchscreenQuestionsMainFinal.pdf). Accessed 22 October 2020a.

**UK Biobank** (n.d.-b) COVID-19 test results data [web site]. (https://biobank.ndph.ox.ac.uk/showcase/exinfo.cgi?src=COVID19_tests). Accessed 18 December 2020b.

**Zhang Q, Bush K, Nolan J, Schnier C, Sudlow C** (2018a) Definitions of Asthma for UK Biobank Phase 1 Outcomes Adjudication - Version 1.0. (https://biobank.ndph.ox.ac.uk/showcase/showcase/docs/alg_outcome_asthma.pdf). Accessed 21 September 2020.

**Zhang Q, Bush K, Nolan J, Schnier C, Sudlow C** (2018b) Definitions of Chronic Obstructive Pulmonary Disease for UK Biobank Phase 1 Outcomes Adjudication - Version 1.0. (https://biobank.ndph.ox.ac.uk/showcase/showcase/docs/alg_outcome_copd.pdf). Accessed 21 September 2020.
